# Diagnosing Early Cardiac Tamponade in Patient with JAK2+ Myeloproliferative Syndrome with Point of Care Ultrasound

**DOI:** 10.24908/pocus.v6i1.14756

**Published:** 2021-04-22

**Authors:** Evan Cameron, Lawrence Istrail

**Affiliations:** 1 Department of Medicine, Inova Fairfax Medical Campus Falls Church, Virginia

**Keywords:** POCUS, cardiac tamponade, JAK2 myeloproliferative syndrome

## Background

Point of care ultrasound (POCUS) is a diagnostic modality growing in popularity and use in medicine in both the acute and chronic management settings. Its utility lies in its non-invasive application, direct user interface, and portability, especially in handheld devices, allowing for quick assessment and triage. Herein is a case of POCUS diagnosing life threatening cardiac tamponade in a patient with a new diagnosis of JAK2+ myeloproliferative syndrome prompting urgent intervention with pericardiocentesis. This case illustrates the utility of POCUS through its ability to serve as a quick diagnostic tool that can hasten intervention for potentially life-threatening conditions.

## Case

A 69-year-old female presented to the emergency room with complaints of shortness of breath for 3 days. She has no known medical history and takes no prescription medications. On arrival she was noted to be in atrial flutter with rapid ventricular response with heart rate in the 160s. Initial management was targeted at controlling the heart rate with diltiazem and digoxin which did not resolve the rapid heart rate. On physical exam, she was tachycardic with bibasilar inspiratory crackles heard on auscultation and had significant hepatosplenomegaly. Her vitals were significant for a pulse of 162 beats per minute and systolic blood pressure >160 mmHg.Preliminary work up revealed significant leukocytosis of 78,000/uL and thrombocytosis of 2,000,000/uL. Bedside handheld ultrasound revealed a circumferential pericardial effusion with evidence of tamponade with right ventricular diastolic collapse, B-lines consistent with pulmonary edema, as well as hepatosplenomegaly (online Video S1). A formal STAT limited echocardiogram was done to further evaluate the extent of the effusion. The formal read stated the patient had a large circumferential pericardial effusion with a swinging heart, borderline diastolic right atrial and right ventricular collapse, as well as inflow variation across the AV valves, though minimal.

Cardiology service was consulted for intervention and the patient was admitted to the Cardiac Intensive Care Unit. Interventional Cardiology service performed a pericardiocentesis the same day of presentation removing 750cc of serosanguinous fluid with a pericardial drain left in place and cytometry sent for analysis. Post procedure, the patient remained significantly tachycardic though her blood pressure improved to the range of 110-130 systolic. After the pericardiocentesis, the patient was continued on diltiazem for atrial flutter rate control which was refractory requiring transesophageal echocardiogram and cardioversion.

The patient was stable after cardioversion and was discharged on metoprolol, amiodarone, and eliquis and was set up with close follow up with cardiology and oncology. She was later confirmed to have a new diagnosis of JAK2+ Myeloproliferative Syndrome.

## Discussion

Point of care ultrasound is a diagnostic modality growing in popularity due to non-invasive nature and quick application. Historically, POCUS has its origins in the trauma setting most notably applied through the Focused Assessment with Sonography in Trauma (FAST Exam) 1. In the acutely ill, outside of trauma, POCUS has shown to be an effective tool to arrive at more accurate etiologies of shock when using POCUS on an immediate basis compared to delayed use 2.Recently, a systematic review showed that POCUS is being used frequently in the primary care setting for diagnostic, screening, and procedural use 3. Additionally, one study has shown that POCUS can influence the diagnostic process in the primary care setting by increasing the confidence of diagnosis and changing management plans 4. In the inpatient setting, one study has shown that POCUS used during ward emergencies was associated with a shorter time to intervention and decreased mortality when compared to a control group 5.

Cardiac tamponade carries a poor prognosis, especially in malignancy, which carries a 1-year mortality rate of 76% 6. Characteristics of cardiac tamponade that can be seen with bedside ultrasound include diastolic collapse of the right ventricle and right atrium, exaggerated respiratory variations in flow across the tricuspid and mitral valves, as well as inferior vena cava plethora 7. On exam and vital signs, patients can present with hypotension, jugular venous distension, and distant heart sounds, otherwise known as Beck’s Triad. Despite the low blood pressure that is characteristic of shock state, some patients with subacute progression of pericardial effusion can be hypertensive. One retrospective study characterizing the hemodynamics of patients with subacute pericardial effusion before and after pericardiocentesis showed that 27% of their study population presented hypertensive and blood pressure decreased after intervention 8. This observation reflects the response that was seen in our patient in that her blood pressure trended towards more normal levels after intervention. Additionally, one systematic review of tamponade hemodynamics has shown that the pooled sensitivity of hypotension in tamponade is 26% 9. Thus, hemodynamics alone are not suggestive of tamponade.

**Figure 1:**
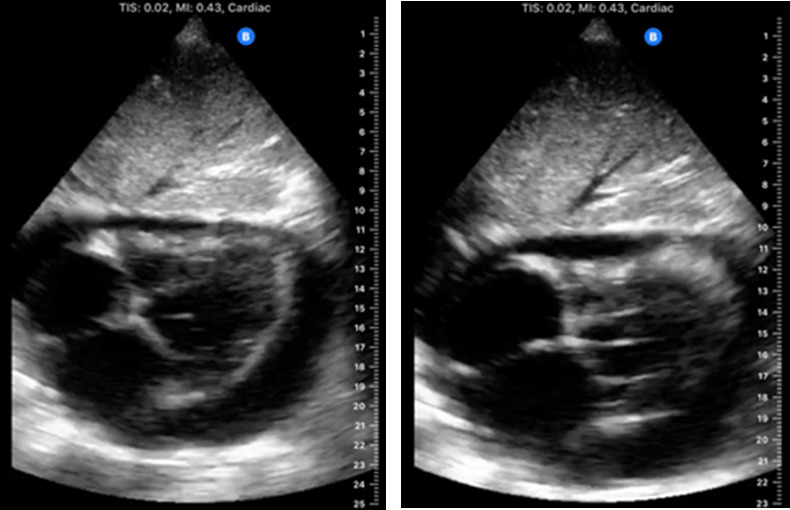
Subcostal Four Chamber View illustrating circumferential pericardial effusion. The two views depict variability in right ventricular wall morphology. Important to note the patient was in atrial flutter with rapid response thus making it difficult to capture diastolic collapse in still frame.

In the case described, POCUS revealed a circumferential pericardial effusion with evidence of cardiac tamponade and pulmonary edema. Though she was not in a florid obstructive shock-like state, the bedside echocardiogram did show evidence of early tamponade suggesting impending cardiovascular collapse. In Video S2, a subcostal 4 chamber view depicts the pericardial effusion with right ventricular collapse. Still-frame photos of the subcostal view show the collapsibility of the right ventricle through the heart cycle (Figure 1). Video S3 shows plethora of the inferior vena cava through the respiratory cycle with a fluttering right atrium. These findings were discussed with Interventional Cardiology which led to escalation of care and early intervention. In the absence of the bedside POCUS exam, the standard of care through the hospital system would have entailed ordering a formal full echocardiogram with reading, cardiology consultation, and hospital admission, all of which takes time that the patient may not have been able to afford clinically as these steps would not always be done in an urgent manner. This route would potentially result in adverse outcomes due to delay in intervention. In this case, the bedside POCUS exam revealed critical physiologic findings that resulted in a hastened escalation of care and intervention which may have saved the patient’s life. This case emphasizes the importance and utility of point of care ultrasound in diagnosis and triage of critical findings.

## Statement of Ethics

The authors certify that verbal patient consent has been obtained for her images and anonymous clinical information to be reported for teaching or in a medical journal. The patient understands that her name, initials, or any identifying information will be kept anonymous and not be published.

## Disclosures

The authors declare no conflicts of interest.

## Supplementary Material 

Video S1 Parasternal Long View revealing circumferential pericardial effusion. The right ventricular wall appears to have abnormal motion throughout the cardiac cycle.

Video S2 Subcostal Four Chamber View again showing the circumferential pericardial effusion. Note the atrial flutter and collapsibility of the right ventricular wall.

Video S3 Subcostal Inferior Vena Cava View demonstrated IVC plethora during inspiration. Note that the collapsibility is blunted during respiration.
